# Kymata Soto Language Dataset: an electro-magnetoencephalographic dataset for natural speech processing

**DOI:** 10.1038/s41597-026-06579-8

**Published:** 2026-01-20

**Authors:** ChenTianyi Yang, Oliver Parish, Anastasia Klimovich-Gray, Cai Wingfield, William D. Marslen-Wilson, Chao Zhang, Alexandra Woolgar, Andrew Thwaites

**Affiliations:** 1 MRC Cognition and Brain Sciences Unit, University of Cambridge, Cambridge, UK; 2 Department for Speech Hearing and Phonetic Sciences, UCL, London, UK; 3 School of Psychology, University of Aberdeen, Aberdeen, UK; 4 Institute for Interdisciplinary Data Science and AI, University of Birmingham, Birmingham, UK; 5 Department of Psychology, University of Cambridge, Cambridge, UK; 6 Department of Electronic Engineering, Tsinghua University, Beijing, China; 7 Shanghai Artificial Intelligence Laboratory, Shanghai, China

**Keywords:** Language, Cortex

## Abstract

The Kymata Soto Language Dataset comprises raw electroencephalographic (EEG) and magnetoencephalographic (MEG) recordings from 15 native Russian speakers and 20 native English speakers as they listened to approximately seven minutes of conversational speech in their respective native languages. Each participant heard the same conversational speech stimulus multiple times (four repetitions for Russian speakers and eight for English speakers). The dataset includes transcriptions of the recordings, along with timestamp annotations for each phoneme and word. Organized according to the Brain Imaging Data Structure (BIDS), this dataset facilitates in-depth research into brain responses to naturalistic speech. To validate the dataset and our preprocessing pipeline, we employed Python-based analyses, revealing consistent low-level loudness perception trends across both language groups. All EEG and MEG data, audio recordings, transcriptions with timestamp annotations, and validation codes are open source, promoting transparency and reproducibility.

## Background & Summary

Understanding how the human brain processes language has long intrigued researchers. While functional Magnetic Resonance Imaging (fMRI) and functional Near-Infrared Spectroscopy (fNIRS) have been widely used, electroencephalography (EEG) and magnetoencephalography (MEG) have gained popularity due to their high temporal resolution (often reaching 1 kHz) and the relatively low cost of EEG. Over the past 15 years, there has been a shift toward studying language comprehension in more naturalistic contexts. Instead of analysing EEG and MEG responses to isolated words or sentences, researchers increasingly focus on continuous speech, such as audio narrations of classic novels like *The Adventures of Sherlock Holmes* and *Alice’s Adventures in Wonderland* (e.g.^1,2^ for EEG^3–7^, for MEG). This approach enables the examination of more complex language processing mechanisms and provides valuable data for applications such as decoding speech from brain recordings^8,9^.

In this paper, we introduce a dataset of simultaneous EEG and MEG (EMEG) recordings from participants listening to multi-participant conversational speech in different languages. This dataset is particularly suited for investigating intra-language linguistic comparisons and cross-language differences in speech perception.

This dataset offers several features that make it particularly valuable for studying cortical language processing. First, it includes data from two distinct language groups: native English speakers listening to English and native Russian speakers listening to Russian. This design allows for meaningful cross-linguistic comparisons (see Table 1).

**Table 1:** Overview of the two data subsets contained in the Kymata Soto Dataset.

Name of Dataset	Name of subset	Audio stimuli	Native language of participant listening to stimuli	Visual stimuli
Kymata SOTO	English-native-English-conversation	BBC podcast of English speakers discussing the history of ice cream	English	Moving dots on a grey background, randomly horizontal oscillation, and also changing in colour. Participants were instructed to look at fixation cross in the centre of the screen.
	Russian-native-Russian-conversation	BBC podcast of Russian speakers discussing the history coffee	Russian	Moving dots on a grey background, separated into quadrants, randomly oscillating in both the horizontal and vertical dimension, and changing in colour. Participants were instructed to look at fixation cross in the centre of the screen.

Second, the dataset features naturalistic conversational speech rather than scripted narrations. Unlike narrated speech, which tends to be slower and more deliberate, spontaneous conversations exhibit more variable pacing influenced by context and individual differences^10,11^. As conversation is the dominant form of spoken communication, studying natural speech is crucial for understanding real-world language processing.

Third, it is a simultaneous EEG and MEG Recording. By combining these two modalities, the dataset has enhanced sensitivity to different sources of neural activity and enables more accurate source reconstruction^12–14^, providing a more comprehensive understanding of neural responses to auditory stimuli.

During the auditory task, participants fixated on a cross while a dynamic dot-field stimulus appeared in their visual periphery, changing colour over time. This ensured consistent visual engagement across trials.

Preregistrations for both studies can be found on OSF.io: English-native-English-conversation^15^; Russian-native-Russian-conversation^16^.

## Methods

### Participants

Fifteen Russian-speaking adults (7 male; 8 female, mean age = 24 years, range = 18–30) and twenty English-speaking adults (11 male; 9 female, mean age = 22 years, range = 18–35) were recruited from the volunteer panel of the Medical Research Council Cognition and Brain Sciences Unit, Cambridge University. Written, informed consent was collected for all participants, and they were paid for their participation. All the participants had normal hearing and no hearing-related disorders. All the participants were native speakers of the language of the dataset. Proficiency in additional languages was not an exclusion criterion for either group of participants. 4 repetitions of the recording were played for the Russian dataset in one recording session, and 8 repetitions (6 repetitions for participant E8) were played for the English dataset in also one recording session. One participant (E3) came for a second session with the same 8 repetitions of the original recording. The studies were approved by the Cambridge Psychology Research Ethics Committee (CPREC; approvals PRE.2010.46 and PRE.2018.101 respectively).

### Stimuli

#### Audio

For both datasets, the audio stimuli were selected from BBC radio-podcast studio discussions, each with a length 400 seconds (6 min 40 sec). The stimuli were presented at a sampling rate of 44.1 kHz with 16-bit resolution. For the English-native-English-conversation dataset, the stimulus is an edited excerpt from an episode of *You’re Dead to Me* titled *The History of Ice Cream*—a three-way discussion (two male, one female) in English concerning the history of ice cream. The original recording is available at https://www.bbc.co.uk/programmes/p0b3kpn9. For the Russian-native-Russian-conversation dataset the stimulus is an edited excerpt from *БибиСева* (BBC Russian Service, 2014), comprising a three-way discussion (two male, one female) in Russian about the history of Colombian coffee. The *БибиСева* archive is no longer publicly hosted, researchers who wish to obtain a copy may contact the BBC directly to inquire about access; however, availability is at the BBC’s discretion and cannot be guaranteed. As the stimuli used in this study were edited versions of these recordings, we have provided a script that automatically reproduce the precise excerpts used in the experiment with the dataset.

To facilitate the use of the dataset in different scenarios, the transcriptions of both recordings were first generated by using Whisper large v2 model^17^ using the default parameters and then corrected manually. We further obtained the timestamp information at both word and phoneme level for each recording by feeding the generated transcriptions to the Kaldi-based Montreal Forced Aligner^18^ with the English MFA dictionary/acoustic model v3_0_0 and Russian MFA dictionary/acoustic model v3_1_0 respectively for the three datasets, which is also being made available.

Both stimuli were selected to provide engaging and naturalistic examples of conversational speech involving multiple participants of different genders. Its content is largely extemporaneous, ensuring that it would not be familiar to participants in the way a well-known public-domain story might be. The topics of the two stimuli were food-related: the history of ice cream for the English conversation and a discussion of coffee for the Russian conversation.

This choice was made for several reasons. First, the topic is sufficiently engaging to maintain participants’ attention throughout the experiment. Second, it is unlikely to elicit strong emotional responses. Third, it is likely to be of interest to a broad range of listeners, including children, should it be required to reuse the stimulus in future studies.

#### Visual

**Fig. 1:**
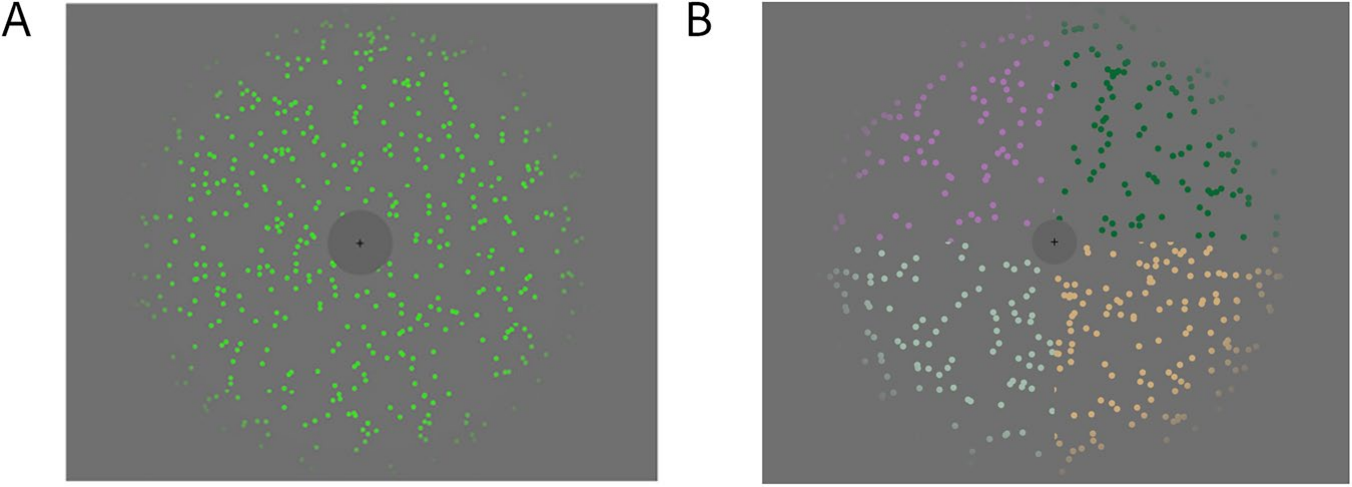
Visual stimuli for the sub-datasets (**A**). Russian-native-Russian-conversation, (**B**). English-native-English-conversation.

For the *Russian-native-Russian-conversation* dataset a pattern of randomly placed colored dots on a grey-masked annular field with a black fixation cross in the center to control eye movements (Fig. 1A). Each dot being 10 × 10 pixels, the central fixation mask being 140 × 140 pixels and the circular edge of the annular field extending to upper and lower edges of the frame, with 100 pixels of linear falloff of the colored dots at the outer edge of the annulus to avoid edge artefacts. Within the annular mask, the horizontal displacement and color of these dots fluctuated pseudo-randomly. The colour moved only in the achromatic and red-green dimensions, and not the yellow-blue dimension. The visual stimulus was 420 seconds long; it started 10 seconds before and ended 10 second after the 400 second auditory stimulus, to avoid the sudden appearance and disappearance of the stimulus during the first and last trial of this auditory stimulus. The stimulus had a refresh rate of 60 Hz at a resolution of 1280 × 1024 pixels.

For the *English-native-English-conversation* dataset, a pattern of randomly placed colored dots on a quadranted grey-masked annular field with a black fixation cross in the center to control eye movements (Fig. 1B). Each dot being 15 × 15 pixels, the central fixation mask being 140 × 140 pixels and the circular edge of the annular field extending to upper and lower edges of the frame, with 100 pixels of linear falloff of the colored dots at the outer edge of the annulus to avoid edge artefacts. Unlike the *Russian-native-Russian-conversation* data set, in which the dots all moved in the same direction and changed color over the entire visual field, in this dataset the visual stimulus was different in each quadrant. Within each quadrant, the horizontal and vertical displacement and color of these dots fluctuated pseudo-randomly. Also, unlike the *Russian-native-Russian-conversation* data, set this dataset’s colors moved through the entire color gamut. The visual stimulus started and ended 10 seconds before and after the stimulus to avoid the sudden appearance and disappearance of the stimulus during the first and last trial.

Each frame of the stimulus (in.png format) is available from the dataset repository (see *data availability*).

The task in all studies was to answer 1–2 multiple-choice questions after each repetition, in the participants native language. These questions served only to keep the subject engaged in the stimulus, and the responses were not recorded.

A dynamic dot field was chosen to facilitate investigation of early visual responses to color and motion (e.g.^19,20^) without eliciting object-detection or other visuosemantic processes. The visual stimulus was not meaningfully synchronised with the audio stimuli, beyond the fact that each stimulus was repeated simultaneously, meaning both could elicit simultaneous stimulus-onset effects.

### Presentation equipment details

The sound was presented through ER3 earpieces with 2.5 m tubes (Etymotic Research, Elk Grove Village, Illinois) controlled with Psychophysics Toolbox^21–23^ in Matlab.

The stimuli were presented on a Panasonic PT-D7700 DLP projector (for the Russian stimulus) and a VPixx ProPiXX (for the English stimulus), both with a refresh rate of 60 Hz. The screen size was 0.5 × 0.4 m. It was placed 1.3 m from the participant, resulting in a viewing angle of 22°. The screen’s white-light luminance was 162 cd/m^2^.

The delays in both sound and visual stimuli presentations are corrected for in the configuration files.

### Procedure

Within each recording session, concurrent EEG and MEG data were recorded for participants, and sampled at 1,000 Hz while they listened to the podcast through ER3 earpieces. The MEG data were recoded using a 306 channel Elekta-Neuromag VectorView (*English-native-English-conversation*) or MEGIN Truix system (*Russian-native-Russian-conversation*) (MEGIN, Espoo, Finland) containing 102 identical sensor triplets (two orthogonal planar gradiometers and one magnetometer) in a hemispherical array situated in a light magnetically shielded room. The EEG data were recorded from 70 Ag-AgCl electrodes placed in an elastic cap (EASYCAP GmbH, Herrsching-Breitbrunn, Germany) according to the 10/20 system, using a nose electrode as reference. Electrocardiogram (ECG), horizontal Electrooculogram (hEOG), and vertical Electrooculogram (vEOG) were also recorded for potential artifact removal during data pre-processing. The stimuli were binaurally presented at approximately 65 dB SPL for both datasets.

Before the experiment, a hearing test was performed to confirm the normal function of the equipment and normal hearing of the participants, and then the opening introduction of the podcast was played for 20 s as a practice session to familiarize the participants with the voices of the speakers and the experimental environment.

The repetitions of the recordings were realized through blocks. Two repetitions were grouped in one block with breaks in between. During these breaks, we talked to the participants to ensure that they felt comfortable before we moved on to the next block. The design of the repetitions was to (i) enable data averaging to enhance signal-to-noise ratio; (ii) give researchers the ability to study individual differences reliably with reference to the variability due to noise across each repetition. The second session for participant E3 was also included to validate analyses of interindividual and intraindividual difference.

To ensure that the participants were paying attention to the recording, we designed two two-alternative forced-choice questions about the content of the podcast after each repetition. We also monitored participants through a camera in the MEG room. If they closed their eyes for prolonged intervals or reported feeling sleepy or unfocused we paused the experiment at the breaks and resumed only when they felt more attentive.

Participants had a T1-weighted Magnetic-Resonance-Imaging (MRI) scan taken, either on a Tim Trio (*English-native-English-conversation*) or Primsa (*Russian-native-Russian -conversation*) 3 T scanner (Siemens, Erlangen, Germany) at the MRC Cognition and Brain Sciences Unit. The scanner used 1-mm isotropic voxels to obtain MRI images using GRAPPA 3D MPRAGE sequence (TR = 2250 ms; TE = 2.99 ms; flip-angle = 9 degrees; acceleration factor = 2) either on the day of their EMEG session or a later depending on the availability of the MRI facility.

Before each EMEG session, the head shape with the position of the EEG electrodes was recorded with a 3-D digitizer (Fastrak Polhemus Inc, Colchester, VA), and co-registered with five head-position indicator (HPI) coils. The head position of participants was checked at the start of each block to ensure that the participant did not move excessively.

### Preprocessing

#### MEG

The MEG dataset and its annotations are shared raw (i.e. not preprocessed) organized according to the Brain Imaging Data Structure (BIDS:^24,25^). Note that researchers wanting to use this data will thus need to pass the data through Signal-Space Separation (SSS^26,27^) and/or maxwell filtering, to remove environmental artefacts.

#### MRI

The individual structural MRI data of the English dataset is provided after being defaced using PyDeface^28^. Due to the wording of the Russian dataset participant consent, the MRI structurals of this dataset are not shared. Researchers wishing to do cortical localisation with these data should use an average cortical mesh such as Freesurfer’s FSaverage^29^.

#### Stimuli

For the auditory stimuli, we include in the dataset: the original audio (‘stimuli/audio/stimulus.wav’), the word-level transcription (‘stimuli/audio/mfa_word.txt’) with the starting time of each word (‘stimuli/audio/mfa_word_stime.txt’), and the phonetic transcription (‘stimuli/audio/mfa_phone.txt’) with the starting time of each phoneme (‘stimuli/audio/mfa_phone_stime.txt’). ‘ < sp > ’ is used in the transcriptions to denote short pauses, and ‘spn’ in the phonetic transcriptions are used for words whose phonetic transcriptions are not available in the pronunciation dictionary.

For the visual stimuli, we save each frame of the patterns on the screen in ‘stimuli/visual/png_frames_over_stimulus’. Each sub-folder inside contains the stimulus by frame in.png format (60 in total) for each second.

#### Triggers

The trigger channel is ‘STI101’. The trigger value ‘1’ is used for the onset of the practice audio stimulus, the trigger value ‘2’ is used to denote each second of the video stimulus, and the trigger value ‘3’ is used for the onset of each repetition of the 400 second audio conversation stimulus file. Button-press trigger values related to the forced-choice questions between runs are between 4000 and 20000, and are not mapped to specific responses.

#### Computing environment

The processing of the present data is based on the free and open-source ecosystem of the neuroimaging community. We used: MNE BIDS^25^ (https://mne.tools/mne-bids) and Bids-Validator (https://github.com/bids-standard/bids-validator)

## Data Records

**Fig. 2:**
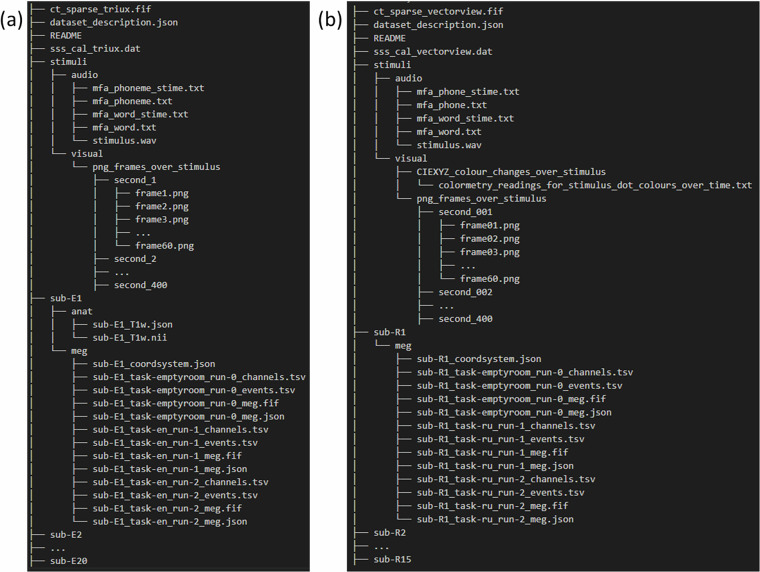
File structure of (**a**) *Russian-native-Russian-conversation* dataset (**b**) *English-native-English-conversation* dataset.

The dataset is organized according to Brain Imaging Data Structure (BIDS) 1.9.0^24^ and publicly available on the Open Science Framework data repository (https://osf.io/qdzgh/)^30^ under a Creative Common Attribution 4.0 International Licence (CC-BY). An image of the folder structure is provided in Fig. 2. The detailed description of the BIDS file system is available at http://bids.neuroimaging.io/. In summary:

- ‘./dataset_description.json’ describes the dataset
- ‘ct_sparse_vectorview.fif’, ‘sss_cal_vectorview.dat’ in the Russian-native-Russian-conversation dataset and ‘ct_sparse_triux.fif’, ‘sss_cal_triux.dat’ in the English-native-English-conversation dataset are the MEG-machine-specific files that can be used to perform Maxwell filtering^26,27^.
- ‘./stimuli/’ contains the original podcasts and visual stimuli used during the experiments
- Each ‘./sub-XXX’ contains the brain recordings of a unique participant. ‘R’ in front of the numbers means native Russian speakers and ‘E’ means native English speakers
- In each participant folder lies the anatomical and the meg data
- ‘en’ in task means listening to English podcast and ‘ru’ in task means listening to Russian podcast
- The dataset can be read directly using MNE-BIDS.

## Technical Validation

We checked that the dataset complies with the standardized brain imaging data structure by using the Bids-Validator (https://github.com/bids-standard/bids-validator).

**Fig. 3:**
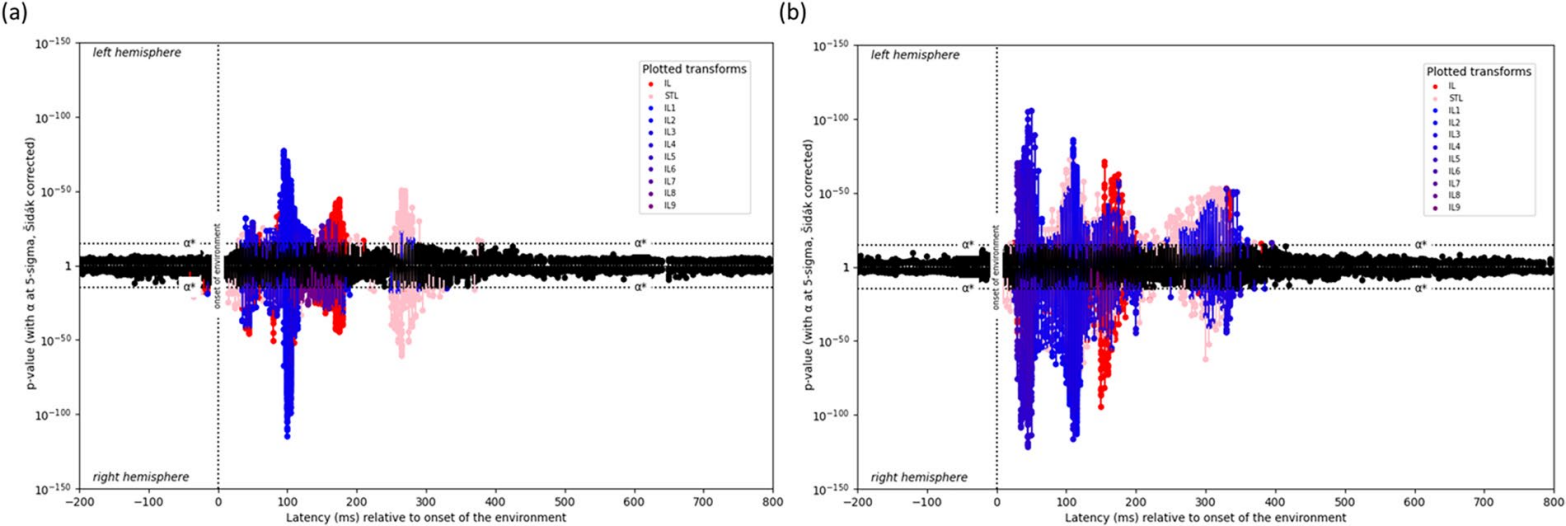
Confirmation that the TVL expression plot is comparable between both (**a**) *Russian-native-Russian-conversation* dataset (**b**) *English-native-English-conversation* dataset.

MEG recordings are subject to large amount of measurement noise, and are thus a challenge to validate empirically. We followed the same analysis procedure as Thwaites *et al*.^31^ and generated the expression plots for three different time-varying loudness (TVL) transformations in the auditory domain – instantaneous loudness, combined instantaneous loudness, and short-term loudness^32^. This preprocessing and validation was performed using the workflows provided by the *kymata-core* software toolbox^33^; TVL transforms of the audio stimuli were computed using a Matlab implementation^34^ of the Moore *et al*. model^32^. These describe the timings and locations at which each of these loudness-related mathematical transformations take place in the cortex. The peaks of the different loudness transforms can be seen to occur at similar latencies for both the *Russian-native-Russian-conversation* and *English-native-English-conversation* dataset (Fig. 3).

The *Russian-native-Russian-conversation* dataset has also been used in numerous existing studies^19,20,31,35^.

## Usage Notes

The following Python code will read the first run of the first subject of the English-native-English-conversation dataset^36^.


from mne_bids import BIDSPath, read_raw_bidsbids_path = BIDSPath(subject = 'E1', task = 'en', run = '1', datatype = 'meg',extension = 'fif', root = 'my/data/path', suffix = 'meg')raw = read_raw_bids(bids_path)


## Data Availability

The dataset is available on OSF at 10.17605/OSF.IO/QDZGH.

## Code availability

Only the open-source MNE BIDS python package^25^ (https://mne.tools/mne-bids) is required to open the files.
